# Equinins as Novel Broad-Spectrum Antimicrobial Peptides Isolated from the Cnidarian *Actinia equina* (Linnaeus, 1758)

**DOI:** 10.3390/md22040172

**Published:** 2024-04-12

**Authors:** Claudia La Corte, Valentina Catania, Mariano Dara, Daniela Parrinello, Mariele Staropoli, Maria Rosa Trapani, Matteo Cammarata, Maria Giovanna Parisi

**Affiliations:** 1 Marine Immunobiology Laboratory, Department of Earth and Marine Sciences (DiSTeM), University of Palermo, Viale delle Scienze, Ed. 16, 90128 Palermo, Italy; claudia.lacorte@unipa.it (C.L.C.); mariano.dara@unipa.it (M.D.); daniela.parrinello@unipa.it (D.P.); mariele.staropoli@unipa.it (M.S.); mariarosa.trapani2@gmail.com (M.R.T.); mariagiovanna.parisi@unipa.it (M.G.P.); 2 NBFC—National Biodiversity Future Center, Piazza Marina 61, 90133 Palermo, Italy; valentina.catania@unipa.it; 3 Department of Earth and Marine Sciences (DiSTeM), University of Palermo, Viale delle Scienze, Ed. 16, 90128 Palermo, Italy

**Keywords:** antibacterial peptides, sea anemones, *Actinia equina*, equinins, Gram-positive and Gram-negative bacteria, minimum inhibitory concentrations (MICs), HPLC

## Abstract

Sea anemones are valuable for therapeutic research as a diversified source of bioactive molecules, due to their diverse bioactive molecules linked to predation and defence mechanisms involving toxins and antimicrobial peptides. Acid extracts from *Actinia equina* tentacles and body were examined for antibacterial activity against Gram-positive, Gram-negative bacteria, and fungi. The peptide fractions showed interesting minimum inhibitory concentration (MIC) values (up to 0.125 µg/mL) against the tested pathogens. Further investigation and characterization of tentacle acid extracts with significant antimicrobial activity led to the purification of peptides through reverse phase chromatography on solid phase and HPLC. Broad-spectrum antimicrobial peptide activity was found in 40% acetonitrile fractions. The resulting peptides had a molecular mass of 2612.91 and 3934.827 Da and MIC ranging from 0.06 to 0.20 mg/mL. Sequencing revealed similarities to AMPs found in amphibians, fish, and Cnidaria, with anti-Gram+, Gram-, antifungal, candidacidal, anti-methicillin-resistant *Staphylococcus aureus*, carbapenemase-producing, vancomycin-resistant bacteria, and multi-drug resistant activity. Peptides 6.2 and 7.3, named Equinin A and B, respectively, were synthesized and evaluated in vitro towards the above-mentioned bacterial pathogens. Equinin B exerted interesting antibacterial activity (MIC and bactericidal concentrations of 1 mg/mL and 0.25 mg/mL, respectively) and gene organization supporting its potential in applied research.

## 1. Introduction

Sea anemones are basically sessile and venomous Cnidarians. Defence mechanisms are also based on molecules involved in predation such as biotoxins including antimicrobial peptides (AMPs). *Actinia equina* (Linnaeus, 1758) is a little-studied anthozoan coelenterate of the Actiniidae family, widespread from the Indo-Pacific to the Mediterranean and the Atlantic, which lives mainly in intertidal zones but is also present up to a few metres deep, as well as capable of remaining out of the sea for hours at a time. It is a small (6–8 cm in height and less than 10 cm in diameter [1]) red to brownish-red sea anemone equipped with stinging tentacles. Its body is cylindrical and has a pedal disc at its base which is wider than the trunk above. The mouth opening is surrounded by about 200 rather short tentacles. During low tide, it often remains out of the water, taking on the appearance of a small ball, or a tomato, with a gelatinous appearance and a hollow in the centre. When fully immersed, it extends its tentacles and assumes a shape similar to that of a flower.

Since the first AMPs were discovered in *Drosophila melanogaster*, they have attracted the attention of scientists for their potential role in human health [2] and as potential defence molecules also in invertebrates, embracing a large range of bacteria and fungi [3,4,5,6]. Generally smaller than 10 kDa, they are characterized by a fast reaction to infection by microorganisms and are also present in tissues exposed to pathogens in invertebrates [6,7,8].

Several AMPs have been identified and characterized in Cnidarians. The first AMP was isolated from the mesoglea of the jellyfish *Aurelia aurita* [9]. This peptide shows, in its amino acid sequence, six cysteines linked together by disulfide bonds and is effective against Gram-positive and Gram-negative bacteria. In *Hydra*, already known AMPs called Hydramacin-1, but also AMPs such as Arminin 1a and Periculin-1, which are exclusive to this taxon, have been identified [10]. One of the objectives of this type of study is to obtain synthetic peptides with antibacterial activity such as the cytolysins sticolisin I and II, identified in *Stychodactyla helianthus*, which do not show significance towards human erythrocytes [11]. In *Anemonia viridis*, our research has revealed that the major pool of neurotoxins are active on sodium and potassium channels and appear to display a dual role as both toxin and antibacterial [12,13].

Many of the AMPs studied to date are amphiphilic with both hydrophobic and hydrophilic surfaces [14]. Their broad antibacterial spectrum and highly selective toxicity make them interesting candidates for safeguarding human health [15,16,17,18]. AMPs obtained from marine organisms are a still underutilized pool of potential broad-spectrum antibiotics [6,9]. Some of the functional motifs, such as amphipathic α-helices and β-sheets, underlying the first antimicrobial molecules have, over time, produced an arsenal of bioactive molecules yet to be studied and discovered but which have characterized the responses, adaptation, and probably the selection of many organisms [19,20].

The goal of our work was to assess the possible bioactive effects of *A. equina* crude tissue extracts, testing their inhibitory capacity versus four distinct bacterial strains, *Escherichia coli*, *Pseudomonas aeruginosa*, *Micrococcus lysodeikticus*, and *Vibrio alginolyticus*, and against the fungus *Candida albicans.* Two peptides extracted, purified, and newly synthesized from the tentacles of *A. equina* were tested against Gram-negative and -positive bacterial strain pathogens to evaluate their antimicrobial activity and were studied through in silico analysis. The cytotoxic efficacy of these crude tissues and neosynthesized peptides towards animal and human erythrocytes was also evaluated.

## 2. Results

### 2.1. Antimicrobial Activity of A. equina Extracts

The antibacterial activity of tentacle and body acid extracts of *A. equina* was preliminarily evaluated in terms of the diameter of the zone of inhibition of free-living bacterial growth against the reference strain pathogens *E. coli*, *M. lysodeikticus*, *V. alginolyticus*, and *P. aeruginosa*. Inhibition zones were observed for tentacle and body extracts tested at 0.5 and 0.25 mg/mL, compared to the negative control. The antimicrobial investigation was continued by focusing on MICs. *A. equina* samples were tested against the relevant pathogens described above, and their antimicrobial activity against free-living microbiological strains was expressed in terms of MIC in mg/mL, as shown in Table 1.

Both extracts had activity towards the bacterial strains tested, with the body acid extracts showing an MIC value of 0.5 mg/mL, and the tentacle acid extracts, an MIC value of 0.25 and 0.5 mg/mL against Gram-negative *E. coli*, *P. aeruginosa*, *V. alginolyticus*, respectively. Tentacle acid extracts showed a remarkable activity against Gram-positive *M. lysodeikticus*, with MIC values of 0.125 mg/mL. No antimicrobial activities were detected in non-acid tentacle and body extracts at the maximum tested concentration of 1 mg/mL against the bacterial strains tested. The MBC was determined for all extracts. Body acid extracts showed bactericidal activity against *E. coli* and *M. lysodeikticus* at the highest concentration of 0.5 mg/mL; the tentacle acid extracts showed bactericidal activity against all three tested bacterial strains at a concentration of 0.5 mg/mL, against *E. coli*, *V. alginolyticus*, and *P. aeruginosa* at 0.25 mg/mL, and at 0.125 mg/mL against *M. lysodeikticus*.

### 2.2. Fractionation of A. equina Tentacle Acid Extracts

As the acid extracts of the tentacles of *A. equina* were the most effective against the bacterial strains tested, they were purified using a silica-based chromatographic column SEP PAK C8 Vac 12cc. The 10, 40, and 80% ACN fractions of the *A. equina* tentacles acquired through SEP PAK were assayed to assess their antibacterial activity against *E. coli*, *V. alginolyticus*, and *M. lysodeikticus* using the modified Kirby–Bauer test. The protein concentration of the same fractions was determined preliminarily, showing a higher quantity of protein in the 40% ACN fraction (7.83 ± 2 mg/mL), compared to the 10% ACN fraction (0.98 ± 0.12 mg/mL), and a minimal amount of protein in the 80% fraction (0.20 ± 0.06 mg/mL). The 40% ACN fraction showed significant activity against all strains tested, up to a dilution of 1/10.

The highest antibacterial activity was towards *M. lysodeikticus*. No antibacterial activities were identified at the maximum tested concentrations for the 10 and 80% ACN fractions of the *A. equina* tentacles.

### 2.3. Reversed-Phase Chromatography

**Figure 1:**
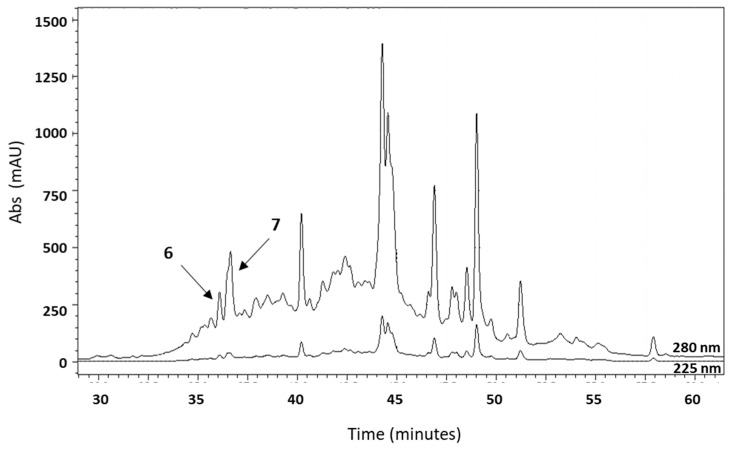
Typical profiles of separation of fraction 6 and fraction 7 of *A. equina* tentacles acquired by reversed-phase chromatography with an ACN gradient from 20 to 60% in acidified water (0.05% trifluoroacetic acid) over 60 min, at a flow rate of 0.5 mL/min. Absorbance peaks were checked at 225 and 280 nm. Arrows show peaks active against *M. lysodeikticus.*

The active molecules of the 40% eluate were subjected to reversed-phase HPLC and eluted with a gradient of 0–60% ACN, generating the chromatogram shown in Figure 1. The graphs exhibit a series of peaks after 30 min of sample input. All peaks were collected, lyophilized, and resuspended in water at a concentration of 2 mg/mL. The antimicrobial activity of all fractions against free-living *M. lysodeikticus* was evaluated and expressed in terms of MIC to detect potential antimicrobial factors. Tests showed high antibacterial activity in peaks between 35 and 37 min, named fractions 6 and 7, assayed and active also for *E. coli*, *S. aureus*, and *C. albicans* at concentrations from 0.06 to 0.20 mg/mL for five distinct experiments.

**Figure 2:**
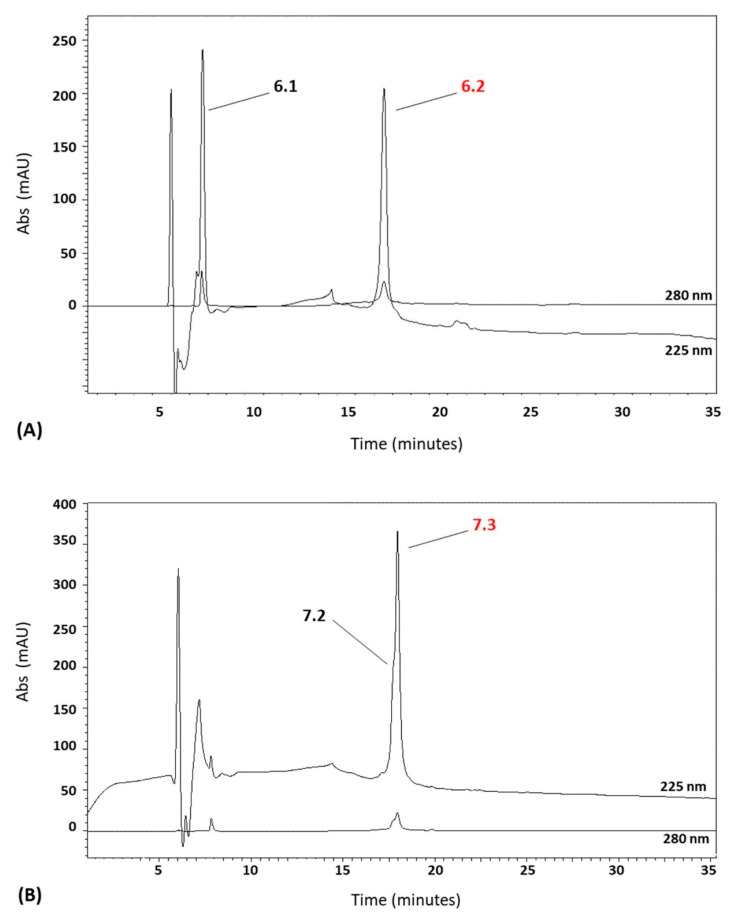
High-performance liquid chromatography (HPLC) profile of the fractions corresponding to peak 6 (**A**) and peak 7 (**B**) of *A. equina* tentacles. Active peaks are in red.

The two fractions showing high antibacterial activity were further purified by reversed-phase chromatography with an ACN gradient from 20 to 60%. Fraction 6 shows two peaks, 6.1 and 6.2 (Figure 2A), and fraction 7 also shows two main peaks, 7.2 and 7.3 (Figure 2B), and there is a single peak that displays antimicrobial activity towards *M. lysodeikticus*, along with *E. coli*, *S. aureus*, and *C. albicans*.

### 2.4. Sequencing

Two novel peptides were identified in acid extracts from *A. equina* tentacles by reversed-phase chromatography. The fractions corresponding to peaks 6.2 and 7.3 of *A. equina* were purified and sequentially sequenced. In order to predict the potential capacity to act as AMPs and to estimate analogies with already described AMPs, bioinformatic analyses were performed. In particular, the “Antimicrobial Peptide Calculator and Predictor” (APD3) tool of the APD was used [21].

Peptides 6.2 and 7.3, named Equinin A and B, showed a sequence similarity percentage between 42 and 37, respectively, with known AMPs identified in marine organisms belonging to amphibians and fish, exhibiting high anti-Gram+, Gram-, antifungal, candidacidal, and anti-methicillin-resistant *Staphylococcus aureus* (MRSA) activity (Table 2). In particular, Equinin A was similar to Palustrin, active against multi-drug resistant (MDR) carbapenemase-producing, MRSA, and vancomycin-resistant bacterial strains, with MIC values ranging from 10–20 to 2560 µg/mL [22]. Equinin B was similar to a novel 40-residue antimicrobial peptide described in the scyphoid jellyfish, *Aurelia aurita*, with activity against Gram-positive *Listeria monocytogenes* (MIC 22.64 µg/mL) and Gram-negative *E. coli* (MIC 7.66 µg/mL) [9]. Aurelin of *A. aurita* has no structural homology with any previously recognised antimicrobial peptides but shows partial similarity with both defensins and K+ channel-blocking toxins of sea anemones and belongs to the ShKT domain family.

The main physico-chemical parameters and characteristics were evaluated using the APD and are described in Table 2.

Equinin A has a molecular weight of 2612.91 Da and is composed of 27 amino acids, while Equinin B has a molecular weight of 3934.827 Da and is composed of 34 amino acids.

The peptides identified in the tentacle acid extract of *A. equina* exhibited a net positive charge, suggesting a more effective role as AMPs compared to anionic peptides. The peptides presented with a hydrophobic ratio ranging from 15 to 19% and high values of Wimley–White whole residues, enhancing their ability to interact with and perturb the bacterial membrane, the primary target of AMPs. Further remarkable parameters are reported in Table 3.

**Figure 3:**
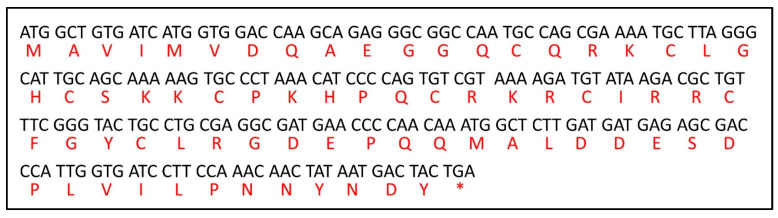
Equinin B nucleotide and amino acid sequence alignment.

The sequences of Equinin B were analysed using SequenceServer 2.0.0.rc7 (Figure 3). The results showed that it was homologous to sequences from *A. equina*. As regards Equinin A, unfortunately, it was not possible to clearly identify the aa sequence and proceed with the same investigations of its structure features.

**Figure 4:**
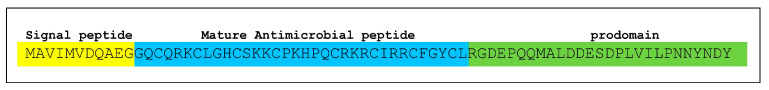
Graphical overview of Equinin B structure predicted by the SignalP 6.0 server. In yellow, the signal peptide region; in light blue, the active AMP; in green, the prodomain.

The SignalP 6.0 server (https://services.healthtech.dtu.dk/services/SignalP-6.0/, accessed on 10 September 2023) was used to predict the presence and location of cleavage sites in the protein. Indeed, Equinin B shows the typical structure of an antimicrobial peptide: the signal peptide region (short N-terminal amino acid sequences that target proteins to the secretory (Sec) pathway in eukaryotes), the active antimicrobial peptide, and a prodomain that usually mediates interaction with other proteins in a complex (Figure 4).

**Figure 5:**
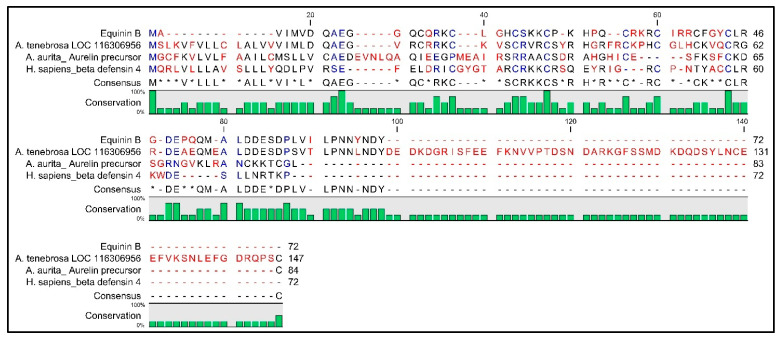
Multiple amino acid alignment between Equinin B and sequences that showed higher similarity obtained from NCBI (default parameters). Conserved amino acids are in blue, isoforms of amino acids are in black and residues revealing fewer properties are in red. Asterisks (*) correspond to ambiguous residues. Hyphens (-) indicate missing residues. Bar plots highlight the amino acid percentage conservation.

The highest identity was observed with uncharacterized protein LOC116306956 of *Actinia tenebrosa* (XP_031572955), consisting of 147 aa, with a high sequence similarity to human beta defensin 4 and kinocidins (Figure 5). The *A. tenebrosa* nucleotide sequence consists of 644 bp; the transcript has 2 exons and is annotated with 9 domains belonging to the calcium-binding protein family, particularly EF-Hand 1 calcium-binding site and Calbindin, and six-EF hand domains.

### 2.5. Antibacterial Activity of Newly Synthesized Peptides: Equinin A and Equinin B

The newly synthesized peptides (Equinin A and Equinin B) were tested to evaluate their antibacterial activity against reference strains of the pathogens *E. coli*, *M. lysodeikticus*, and *V. alginolyticus*. Antimicrobial activity against free-living strains (i.e., planktonic cell cultivation) was expressed in terms of MIC. The results are illustrated in Table 4. Among the peptides evaluated (Equinin A and B), only Equinin B had antimicrobial activity at the tested concentration. In particular, Equinin B was active against all bacterial strains tested at the maximum concentration used of 1 mg/mL up to 0.25 mg/mL. Equinin A did not show antimicrobial activity at the maximum tested concentration of 1 mg/mL.

### 2.6. Haemolytic Activity

Lytic activity towards human erythrocytes of the AB+ and 0+ blood groups was tested both for the neosynthesized peptides (Equinin A and B) and the crude extracts that had demonstrated increased antimicrobial activity.

**Figure 6:**
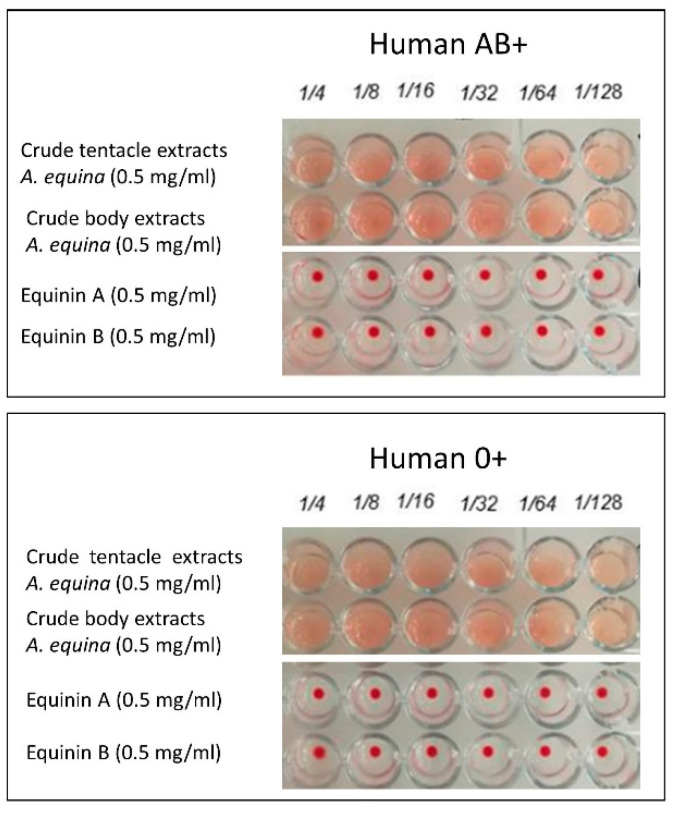
Haemolytic activity of crude extracts and synthesized peptides of *A. equina*.

Lytic capacity was investigated by dilution of the crude and synthesized samples of *A. equina.* Despite the former clearly showing lysis even at major dilutions, this was not observed for the peptides (Figure 6).

### 2.7. AMP Predictions through In Silico Analysis

Significant biological parameters of Equinin A and B were analysed to clarify their potential antimicrobial and antibiofilm activity, cell penetrating peptide potential, and the presence of cleavage sites. According to online webserver predictions, two synthetic derivative peptides exhibited high probability (in percentage) of acting as good antimicrobial and antifungal peptides, showing activity with a value >0.5 (Table 5). Specifically, Equinin B revealed significant antimicrobial and antiviral activity compared to Equinin A. Moreover, Equinin B showed high antiviral activity, unlike Equinin A, which showed little antiviral activity (value < 0.5). Equinin A was also characterized by resistance to proteolysis by pepsin.

**Figure 7:**
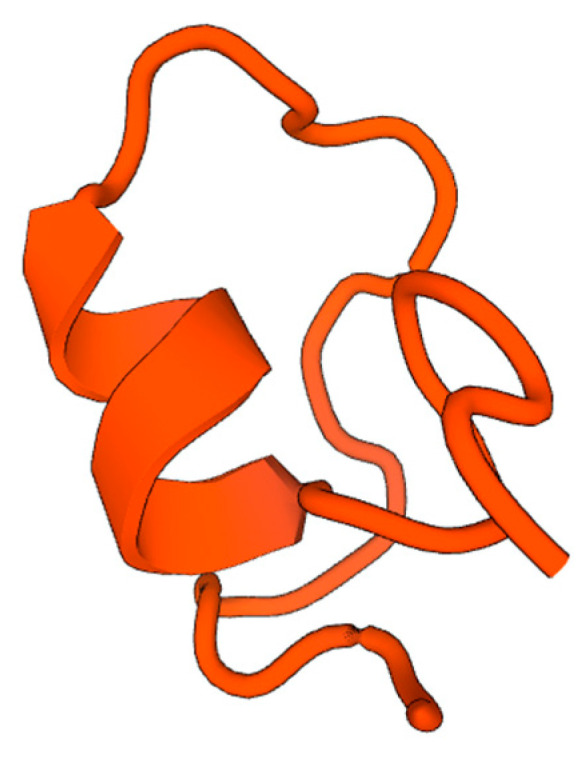
Equinin B predicted 3D structure based on Aurelin sequence. Sequence homogeneity 43.33%. The model has one α-helix.

Only the 3D structure of Equinin B was determined on SWISS-MODEL, since the length of the amino acid sequence of Equinin A was too short for analysis. The obtained three-dimensional structure was modelled on the sequence of the Aurelin antimicrobial peptide (accession: Q0MWV8; sequence identity = 43.33%). The model comes with one α-helices, structures typically ascribable to certain types of small AMPs, and it suggests a possible, although low, similarity reliability of the proposed model (Figure 7).

## 3. Discussion

Many marine organisms, including invertebrates, produce specific compounds useful for protection or for making the organism venomous, unpleasant, or irritating. The boundary between toxins and molecules useful for medical and pharmacological purposes is labile. In fact, in Cnidaria, over 3000 bioactive molecules have been described and are recognized as having several biological interactions [23]. Most of these organisms do not cause irreparable damage to human health, although some Australian species are known to be highly dangerous. Studies impute the poisonousness to proteins present in the venom with phospholipase activity capable of compromising the normal cellular activities by forming pores, provoking alterations in ion exchanges and in membrane permeability in the excitability of cells and neuromuscular transmission of signals and reactive oxygen species (ROS) production [24,25,26]. Traditional therapies have employed several of these substances to treat hypertension, pulmonary, urogenital, dermatological, neurological, and haematological illnesses [27,28,29]. The channel inhibitors might be employed to investigate processes beyond sodium transport and produce biocides. On the other hand, voltage-gated potassium channel toxins have been tested to treat multiple sclerosis and other autoimmune disorders [30,31].

Here, the presence of sources of bioactive molecules and their biocompatibility were investigated in the anemone *A. equina* to highlight their possible uses for biotechnological or therapeutic applications.

Indeed, two possible antimicrobial peptides were identified by biological assays from the crude anthozoan extracts. Following purification by the HPLC technique and subsequent sequencing, analyses were carried out on the structure and possible action mechanisms of the sequences thus obtained. In silico analysis showed Equinin B to have a higher theoretical mass and sequence length than Equinin A, but, above all, the amount of the positively charged amino acids lysine (Lys: 18%), arginine (Arg: 15%), and histidine (His: 6%) make it a cationic peptide, which is one of the characteristics of a good antimicrobial peptide. As the Boman index was higher than 2.48, it also suggested a strong capability of the molecule to bind with other membranes or proteins, thus interacting with them. Due to the fact that the sequence contains an even number of cysteines (Cys), this explains the possibility of forming a beta structure similar to defensin bound with disulphide bonds (~16–60 AA residues), helical structures with S-S bonds, or multiple thioether bonds, as in lantibiotics, due to the high serine (Ser) content.

For all these reasons, the differences found between the two peptides suggest that the action of Equinin A may be more limited, and the concentrations required for it to be effective may need to be higher. Further investigations into the different behaviour of the purified extract compared to the synthesised one are necessary, and the possibility of carrying out further purification and sequencing is the key to better understanding the possible characteristics, functions, and action mechanisms of this potential AMP, also given its similarity with peptides already in the database whose 3D structure is unknown.

Studies support the idea that the production of AMPs by hydrozoans such as *Hydra magnipapillata* can act as an ancient host defence component, given the lack of protective layers and mobile phagocytes that help avoid pathogenic infections [10]. Bosch et al. have, in fact, proved that innate immune responses are mediated by epithelial layers and are based on the unconventional signalling pathway of the Toll-Like Receptor. Moreover, *Pseudomonas aeruginosa* affects the morphology of ectodermal cells, which become round and produce granules typical of immune cells of organisms belonging to other phyla. Hydramacin-1 (purified from *Hydra*), has been proven to have microbiocidal activity inducible by microbial substances, e.g., dose-dependent lipopolysaccharide, and is capable of inhibiting the growth of some Gram-positive (*Bacillus megaterium*) and Gram-negative (*E. coli, Klebsiella oxytoca*, and *Klebsiella pneumoniae*) bacteria [10]. Similarly to what has been observed in *A. equina*, Arminin identified in *H. magnipapillata* did not show toxicity to human erythrocytes (a crucial indicator of biocompatibility) and is characterized by a negative charge with a highly conserved N-terminal region and a poorly preserved positively charged C-terminal region [32]; Arminin 1a-C, an antimicrobial peptide with an α-helical structure from ancient metazoan *Hydra*, efficaciously suppressed the viability of leukaemia cell lines, whether or not they were MDR or sensitive, and independently of the cell lines considered, also exhibiting a distinct discernment between noncancerous and cancerous cell lines [33]. The relevance of the structural characteristics of the bioactive molecules in defining their properties suggests that the different MICs needed to prompt an antibacterial response between the purified *A. equina* AMPs and the synthesized peptide may be attributable to the conformational needs of the molecule itself. This would also explain the incomplete overlap of the Equinin B amino acid sequence with homologous sequences in the *A. equina* genome. Its antibacterial activity has been proven to impair the growth of *E. coli*, *B. megaterium*, and *S. aureus*, and ultra-structural analysis highlighted that Arminin 1 causes a rupture of the bacterial cell membrane. An examination of the antimicrobial characteristics of extracts from Red Sea soft and stony (scleractinian) corals revealed that the majority of soft corals (83%) inhibited the growth of the marine bacteria *Arthrobacter* sp. (two strains) and barely affected the growth of *Vibrio* sp., whereas stony corals had little or no antimicrobial activity. This means that soft and hard corals could have developed different means to deal with pathogens, and this could be due to the different selective pressure and to the environment, as well as to their position occupied in the phylogenetic tree [34]. The potential applications of these compounds are endless. Indeed, antimicrobial peptides are more advantageous to use compared to conventional antibiotics, both for their efficiency against antibiotic-resistant bacteria and for the intrinsic characteristics of AMPs. They can be synthesized at a relatively low cost and with a weak risk of sensitization, ensuring their potential for extensive use in the medical field. Additionally, due to their short lifespan, they do not persist in the environment, making them less available for aquatic organisms [35]. To date, some proteases and AMPs have been purified by our group from tissue extracts of the Mediterranean sea anemones *A. equina* and *A. viridis* and used in the restoration of cultural heritage as antifungals and for the cleaning of artworks [36,37]. Moreover, research on the mucus of *A. equina* evidenced the presence of compounds with antimicrobial and lysozyme-like activities. As a result, it acts as an antifouling substance, counteracting the bacterial colonization of structures. This could be a good starting point for Equinin B as the in silico analysis showed a possible antibiofilm activity. It has been discovered that pH, ionic strength, and temperature affect the mucus’s ability to function. Lastly, *A. equina* extracts present an intriguing prospect for future improvements in the fight against pathogenic bacteria due to their action versus *M. lysodeikticus*, also supported by the excellent results achieved at 37 °C [38].

## 4. Materials and Methods

### 4.1. A. equina Collection and Extract Preparation

*A. equina* specimens were collected in the area of Capo Gallo (northwest Sicily, Italy).

In total, about 80 samples were collected. Pools of 6 animals were used for each purification procedure for peptide synthesis and 6 animals for estimate antimicrobial activity; three biological replicates and three technical replicates for each were therefore carried out.

**Figure 8:**
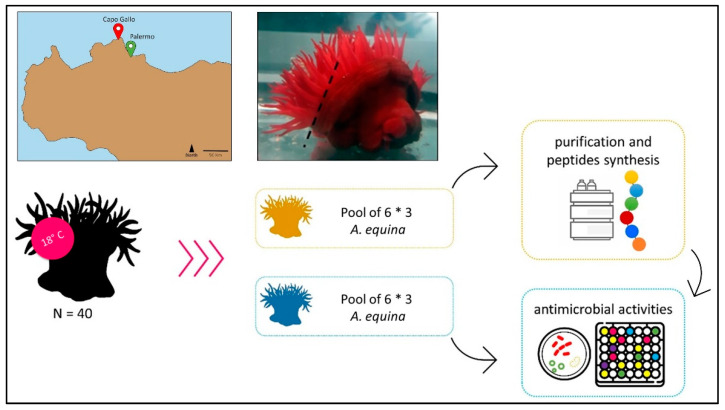
The map shows the sampling site. The samples were collected from the B zone of the Capo Gallo Marine Protected Area, Sicily, Italy. A schematic drawing of the experimental plan and the cutting area (black segment) to separate tentacles from the body is also represented.

The specimens were carried to the laboratory where they were kept in tanks at a temperature of 18 °C (Figure 8). The tentacles were separated from the animal’s body with forceps and were suspended in Tris-buffered solution (TBS; 150 mM NaCl, 10 mM Tris-HCl, pH 7.4), then homogenized in Ultra-Turrax for 5 min on ice; for acidic samples, 10% acetic acid was added. The samples thus obtained were sonicated (Branson Model B15, Danbury, CT, USA) 3 times for 30 s and subsequently centrifuged at 21,000× *g* for 20 min at 4 °C. The protein concentration of the obtained samples was measured by evaluating the absorbance at 595 nm [39]. When necessary, the samples were lyophilized and suspended in TBS at the standard concentration of 0.5 mg/mL.

### 4.2. Microbial Strains

Reference strains of *Escherichia coli* ATCC 25922, *Micrococcus lysodeikticus* ATCC 4698, *Vibrio alginolyticus* ATTC 17749, *Staphylococcus aureus* ATCC 25923, *Pseudomonas aeruginosa* ATCC 15442, and *Candida albicans* ATCC 10231 were used in the evaluation of antimicrobial properties. The modified Kirby–Bauer test was performed, and minimum inhibitory concentrations (MICs) and minimum bactericidal concentration (MBC) were defined. The bacterial strains were cultured aerobically in Poor–Broth nutrient medium (1% Bactotrypton, 0.5% NaCl, *w*/*v*) and tryptic soy agar (TSA) or Luria–Bertani medium (LB). The bacterial cultures were incubated at 37 °C overnight. Fungal strain C. albicans was cultured in Sabouraud broth (BS) or agar plates at 37 °C overnight.

### 4.3. Evaluation of Antibacterial Activity

The antimicrobial activity of acid extracts from the tentacles and body of *A. equina* was investigated by the modified Kirby–Bauer test [40,41] versus *E. coli*, *M. lysodeikticus*, and *V. alginolyticus*. A bacterial suspension from each overnight culture was prepared in NaCl 0.9% (*v*/*v*), to a cell density of approximately 10^5^ CFU/mL, then dispensed onto LB plates. Wells of 6.3 mm diameter were shaped in agarose gel and each filled with 10 μL of acid extracts from the tentacles and body of *A. equina* at concentrations of 5 and 2.5 mg/mL. LB agar plates were incubated at 37 °C overnight. After incubation, the presence of inhibition zone was evaluated.

### 4.4. Determination of MICs and MBC

MICs were established using the microdilution method. Serial dilutions of each extract were made in Poor–Broth nutrient medium in a 96-well plate, starting from a stock solution of 0.5 mg/mL in TBS. A series of concentrations of each acid extract, both from body and tentacle, ranging between 0.5, 0.25, and 0.125 mg/ mL, were tested against the reference bacterial strains *E. coli*, *M. lysodeikticus*, *P. aeruginosa*, and *V. alginolyticus*. A bacterial suspension was prepared, initiating from a culture grown at 37 °C for 24 h on TSA in 0.9% NaCl to 106 colony-forming units (CFUs)/mL, and 80 μL of the suspension were added to each well. A positive control to check bacterial growth, consisting of bacterial strains in the medium without extract to evidence bacterial growth and a negative control to check medium sterility, represented by the medium without inoculum, were also included in the 96-well plate. Additionally, a substance control to estimate absorbance, consisting only of the substance solution without bacterial inoculum, was also added.

The 96-well plates were incubated at 37 °C for 16 h, and MICs were read by a Tecan microplate reader (Infinite 200 M) as the lowest concentration of extract whose optical density (OD), read at 570 nm, was comparable to the negative control wells (only broth). Antifungal activity against C. albicans ATCC 10231 were investigated in terms of MICs by using the micro-method described above, using Sabouraud broth (BS) as growth media. MBC was obtained by plating the contents of wells with no growth of bacteria onto TSA Petri dishes. The minimal fungicidal concentration was detected as described using Sabouraud agar plates for growth of fungal strain. The MBC was specified as the lowest concentration of substance that enabled microbial growth up to a maximum of three colonies after 16 h of incubation at 37 °C.

### 4.5. Solid Phase Extraction and Reversed-Phase HPLC Purification

The acidic extracts of the tentacles were loaded onto Sep-Pak C8 Vac cartridges (Waters Associates, Milford, MA, USA) previously equilibrated with acidified water (0.05% trifluoracetic acid in ultrapure water); the trials were carried out in triplicate. The fractions thus obtained were freeze-dried and subsequently reconstituted with UPW. Most of the antibacterial activity was found in the 40% fraction, which was then subjected to reversed-phase high-performance liquid chromatography (HPLC) on an Interchrom UP5ODB-25QS (250 × 4.6 mm) C18 silica column. Samples of 50 μL were eluted with a linear gradient of 0–60% acetonitrile (ACN) in acidified water for 60 min at a flow rate of 0.5 mL/min. The fractions were collected in tubes equivalent to the absorbance peaks identified at both 280 and 225 nm, subsequently lyophilized and reconstituted in UPW. The active fractions were then re-purified on the same column but with a linear gradient from 20 to 60% ACN under the same conditions. As previously described, each collected peak was freeze-dried, reconstituted at a constant concentration, and tested for anti-M. lysodeikticus, anti-S. aureus, anti-V. alginolyticus, and anti-C. albicans activity by the modified Kirby–Bauer test. The protein concentration of the several ACN fractions for body and tentacles are shown in Table 6.

### 4.6. Amino Acid Sequencing

The purified peptides were deposited and dried on a glass fibre disc pre-coated with Biobren Plus (Applied Biosystems, Waltham, MA, USA) and sequenced on Procise P494 (Applied Biosystems, Waltham, MA, USA) by multiple rounds of Edman degradation online with a PTH amino acid HPLC analyzer (model 785A). The amino acid sequence of the peptides was the sequence in the public UniProtKB/Swiss-Prot databases using the Blast tool from the NCBI Protein Blast website.

### 4.7. Peptide Synthesis

Two peptides were synthesized by GenScript, Biotech Corporation (Somerset, NJ, USA) obtained using FlexPeptide^TM^ Synthesis technology. The quality and purity of each peptide (≈98%) was guaranteed by the company. The powdered peptides were kept at −20 °C for storage. They were dissolved in water to obtain a stock solution of 2 mg/mL. Working solutions of 0.5 mg/mL were prepared for each peptide in TBS, and antimicrobial activity was evaluated by MIC assay as described above.

### 4.8. Haemolytic Activity

Haemolytic activity was tested against human erythrocytes groups AB+ and 0+. The blood samples were provided by the Immunohaematology and Transfusion Medicine Centre of Palermo. The erythrocytes were washed three times in phosphate-buffered saline (PBS), centrifuged at 500× *g* for 5 min. at 4 °C, and washed twice in PBS. Suspensions of erythrocytes at 1% (*v*/*v*) in PBS Gel (6 mM KH_2_PO_4_, 0.11 M NaCl, 30 mM Na_2_HPO_4_, 0.1% *w*/*v* gel, pH 7.4) were used to test the lysis of erythrocytes by tentacle and body acid extracts of A. equina and synthetic peptides. For the microplate assay, 25 μL of samples or serial (two-fold) dilution were mixed with an equal volume of the erythrocyte suspension in PBS Gel in 96-well round-bottom microtiter plates. After 1 h incubation at 37 °C, lytic activity was evaluated and expressed as described by [42]. A positive control with 25 μL of distilled water and 25 μL of erythrocyte suspension and a negative control consisting of 25 μL of PBS Gel and 25 μL of erythrocyte suspension were prepared to compare the activity of the samples on erythrocytes.

### 4.9. In Silico Analysis

The prediction databases used were as follows: APD, the Antimicrobial Peptide Database; CAMP, Collection of Antimicrobial Peptides; and AMPA [43,44,45,46]. Two characteristic indices have been determined: the Wimley–White hydrophobicity scales and the Boman index [43]. Normally employed to predict the transmembrane α-helices of membrane proteins [47], a low value (≤1) of Boman index denotes that the peptide has an elevated antibacterial activity [3]. All this in silico analysis allowed us to verify which of the two purified equinins had a higher probability of being an antimicrobial factor.

The reliability of the predicted 3D structure was assessed using the default method QMEANDisCo global score parameter (=0.36 ± 0.12). The score is expressed with a number between 0 and 1; a higher number indicates a greater reliability of the forecast. This consists of a single model method combining statistical potentials and agreement terms with a distance constraints (DisCo) score. DisCo estimates consistencies of pairwise CA-CA distances from a model with constraints extracted from homologous structures. All scores are combined using a neural network trained to predict per-residue lDDT scores [48,49].

Physico-chemical parameters, biological properties, and the antibacterial and antifungal potential of synthetic peptides were obtained using the APD (APD3; https://aps.unmc.edu/, (accessed on 10 September 2023), dPABBs (https://ab-openlab.csir.res.in/abp/antibiofilm/feature.php, (accessed on 10 September 2023), CellPPD (http://crdd.osdd.net/raghava/cellppd/, (accessed on 10 September 2023), iAMPpred tool (http://cabgrid.res.in:8080/amppred, (accessed on 10 September 2023), and the SignalP—6.0 server (https://services.healthtech.dtu.dk/services/SignalP-6.0/, (accessed on 10 September 2023), respectively.

## 5. Conclusions

In this work, potential AMPs were purified and characterized from tentacle extracts of the anthozoan *A. equina*. These showed broad-spectrum antimicrobial activity towards Gram-positive and -negative bacteria and fungi and were non-toxic for human erythrocytes. The purified extracts were sequenced, and the peptides synthesized. Moreover, the gene sequence and protein and domain organisation were analysed. The outcomes suggest that Equinin B is an optimum candidate as a new AMP, and the in silico analysis opens up future perspectives such as the possibility of performing antibiofilm and antifouling assays or using this compound in aquaculture as an alternative to the use of antibiotics for preventing pathogens in commercial species. It may be desirable, given the non-toxicity of these peptides to human erythrocytes, to assess the antiviral and anticancer activity of this new compound. Another possibility, as has already been performed for other antimicrobial peptides, would be producing new antibodies and primers for the microlocalisation, distribution, and expression of Equinin B in species more or less phylogenetically close to *A. equina*. Finally, as regards Equinin A, driven by the interesting results of in silico analyses, further purifications and peptide syntheses are ongoing.

## Figures and Tables

**Table 1:** Antimicrobial activity of tentacle and body acid extracts of *A. equina*, tested against free-living reference bacterial strains. (-) No antimicrobial activity.

Minimum Inhibitory Concentration (MIC) in mg/mL		
	Tentacle Acid Extracts	Body Acid Extracts
*E. coli* ATCC 25922	0.25	0.5
*M. lysodeikticus* ATCC 4698	0.125	0.5
*V. alginolyticus* ATCC 17749	0.5	0.5
*P. aeruginosa* ATCC 15442	0.25	-

**Table 2:** Identified peptides of *A. equina* tentacles and similarity with already described AMPs.

**Tentacle Extracts**	**Peptide**	**Identified Sequences**	**Percentage** **Similarity with Already Described AMPs**	**Activity** **of AMPs with Better Similarity**	**References**
	Equinin A	AVDKGGGKAEKKDGNRKKKLAGGEGGG	42.42% RaCa-1, Palustrin (frogs, amphibians)	Anti-Gram+ & Gram-	[22]
	Equinin B	GQCQRKCLGHCSKKCPKHPQCRKRCIRRCFGYCL	37.78% Aurelin (Jellyfish, Cnidaria)	Anti-Gram+ & Gram-	[9]

**Table 3:** Principal physico-chemical parameters of potential AMPs from tentacle acid extracts of *A. equina*.

	Equinin A	Equinin B
Peptide residues	27	34
Monoisotopic Theoretical mass (Da)	2612.91	3934.827
Net charge	+4	+11.5
Wimley-White whole residues (kcal/mol)	14.77	8.97
Hydrophobic ratio (%)	19	32
Protein-binding potential Boman index (kcal/mol)	2.54	3.24
GRAVY (i.e., the grand average hydropathy value of the peptide)	−1.46	−1.11

**Table 4:** Antimicrobial activity of synthesized peptides against reference bacterial free-living strains.

Minimum Inhibitory Concentration (MIC) in mg/mL		
	Equinin A	Equinin B
*E. coli* ATCC 25922	>1	0.25
*M. lysodeikticus* ATCC 4698	>1	0.25
*V. alginolyticus* ATTC 17749	>1	0.25

**Table 5:** In silico properties predicted using the following tools and servers: dPABBs (http://ab-openlab.csir.res.in/abp/antibiofilm/protein.php, (accessed on 10 September 2023); CellPPD (http://crdd.osdd.net/raghava/cellppd/submission.php, (accessed on 10 September 2023); iAMPpred (http://cabgrid.res.in:8080/amppred, (accessed on 10 September 2023).

Properties	Equinin A	Equinin B
CPP (cell penetrating peptides)	Not CPP	Not CPP
Antibacterial activity	0.91	1
Antifungal activity	0.8	0.99
Antiviral activity	0.37	0.96
Antibiofilm activity	inactive	active
Degradation by trypsin	Yes	Yes
Degradation by pepsin (pH = 1.3)	No	Yes
Degradation by pepsin (pH > 2)	No	Yes

**Table 6:** Protein concentration of ACN fractions of *A. equina* extracts.

Extracts (mg/mL)	Fractions		
	10%	40%	60%
*Tentacles*	0.98	7.83	0.2
*Body*	1.29	3.49	0.13

## Data Availability

The authors declare that the data supporting the findings of this study are available within the paper. Raw data is available from the corresponding author upon reasonable request.

## References

[1] Fischer W., Bauchot M.L., Schneider M. (1987). Fiches FAO d’ Identification Des Espèces Pour Les Besoins de La Pêche. (Revision 1). Méditerranée et Mer Noire. Zone de Pêche 37. Volume I. Végëtaux et Invertébrés.

[2] Steiner H., Hultmark D., Engström Å., Bennich H., Boman H.G. (1981). Sequence and Specificity of Two Antibacterial Proteins Involved in Insect Immunity. Nature.

[3] Boman H.G. (2003). Antibacterial Peptides: Basic Facts and Emerging Concepts. J. Intern. Med..

[4] Bulet P., Stöcklin R., Menin L. (2004). Anti-microbial Peptides: From Invertebrates to Vertebrates. Immunol. Rev..

[5] Wang W., Gao J., Tong P., Chen H. (2017). Antimicrobial Peptides: Action Mechanism, Application and Improvement Strategy. Chin. J. Anim. Nutr..

[6] Wu R., Patocka J., Nepovimova E., Oleksak P., Valis M., Wu W., Kuca K. (2021). Marine Invertebrate Peptides: Antimicrobial Peptides. Front. Microbiol..

[7] Bartlett T.C., Cuthbertson B.J., Shepard E.F., Chapman R.W., Gross P.S., Warr G.W. (2002). Crustins, Homologues of an 11.5-KDa Antibacterial Peptide, from Two Species of Penaeid Shrimp, *Litopenaeus vannamei* and *Litopenaeus setiferus*. Mar. Biotechnol..

[8] Chisholm J.R.S., Smith V.J. (1992). Antibacterial Activity in the Haemocytes of the Shore Crab, *Carcinus maenas*. J. Mar. Biol. Assoc..

[9] Ovchinnikova T.V., Balandin S.V., Aleshina G.M., Tagaev A.A., Leonova Y.F., Krasnodembsky E.D., Men’shenin A.V., Kokryakov V.N. (2006). Aurelin, a Novel Antimicrobial Peptide from Jellyfish *Aurelia aurita* with Structural Features of Defensins and Channel-Blocking Toxins. Biochem. Biophys. Res. Commun..

[10] Bosch T.C.G., Augustin R., Anton-Erxleben F., Fraune S., Hemmrich G., Zill H., Rosenstiel P., Jacobs G., Schreiber S., Leippe M. (2009). Uncovering the Evolutionary History of Innate Immunity: The Simple Metazoan Hydra Uses Epithelial Cells for Host Defence. Dev. Comp. Immunol..

[11] Tejuca M., Dalla Serra M., Ferreras M., Lanio M.E., Menestrina G. (1996). Mechanism of Membrane Permeabilization by Sticholysin I, a Cytolysin Isolated from the Venom of the Sea Anemone *Stichodactyla helianthus*. Biochemistry.

[12] Trapani M.R., Parisi M.G., Toubiana M., Coquet L., Jouenne T., Roch P., Cammarata M. (2014). First Evidence of Antimicrobial Activity of Neurotoxin 2 from *Anemonia sulcata* (Cnidaria). Invertebr. Surviv. J..

[13] Nicosia A., Mikov A., Cammarata M., Colombo P., Andreev Y., Kozlov S., Cuttitta A. (2018). The *Anemonia viridis* Venom: Coupling Biochemical Purification and Rna-Seq for Translational Research. Mar. Drugs.

[14] Tincu J.A., Taylor S.W. (2004). Antimicrobial Peptides from Marine Invertebrates. Antimicrob. Agents Chemother..

[15] Boman H.G. (1998). Gene-Encoded Peptide Antibiotics and the Concept of Innate Immunity: An Update Review. Scand. J. Immunol..

[16] Lehrer R.I., Ganz T. (1999). Antimicrobial Peptides in Mammalian and Insect Host Defence. Curr. Opin. Immunol..

[17] Hancock R.E.W., Scott M.G. (2000). The Role of Antimicrobial Peptides in Animal Defenses. Proc. Natl. Acad. Sci. USA.

[18] van der Does A.M., Hiemstra P.S., Mookherjee N. (2019). Antimicrobial Host Defence Peptides: Immunomodulatory Functions and Translational Prospects. Antimicrobial Peptides: Basics for Clinical Application.

[19] Kumar P., Kizhakkedathu J.N., Straus S.K. (2018). Antimicrobial Peptides: Diversity, Mechanism of Action and Strategies to Improve the Activity and Biocompatibility in Vivo. Biomolecules.

[20] Smith V.J., Desbois A.P., Dyrynda E.A. (2010). Conventional and Unconventional Antimicrobials from Fish, Marine Invertebrates and Micro-Algae. Mar. Drugs.

[21] Punginelli D., Catania V., Vazzana M., Mauro M., Spinello A., Barone G., Barberi G., Fiorica C., Vitale M., Cunsolo V. (2022). A Novel Peptide with Antifungal Activity from Red Swamp Crayfish *Procambarus clarkii*. Antibiotics.

[22] Li C., Sutherland D., Hammond S.A., Yang C., Taho F., Bergman L., Houston S., Warren R.L., Wong T., Hoang L.M.N. (2022). AMPlify: Attentive Deep Learning Model for Discovery of Novel Antimicrobial Peptides Effective against WHO Priority Pathogens. BMC Genom..

[23] Trapani M.R., Parisi M.G., Maisano M., Mauceri A., Cammarata M. (2016). Old Weapons for New Wars: Bioactive Molecules From Cnidarian Internal Defense Systems. Cent. Nerv. Syst. Agents Med. Chem..

[24] Lassen S., Helmholz H., Ruhnau C., Prange A. (2011). A Novel Proteinaceous Cytotoxin from the Northern Scyphozoa *Cyanea capillata* (L.) with Structural Homology to Cubozoan Haemolysins. Toxicon.

[25] Morabito R., Condello S., Currò M., Marino A., Ientile R., La Spada G. (2012). Oxidative Stress Induced by Crude Venom from the Jellyfish *Pelagia noctiluca* in Neuronal-like Differentiated SH-SY5Y Cells. Toxicol. Vitr..

[26] Destoumieux-Garzón D., Rosa R.D., Schmitt P., Barreto C., Vidal-Dupiol J., Mitta G., Gueguen Y., Bachère E. (2016). Antimicrobial Peptides in Marine Invertebrate Health and Disease. Philos. Trans. R. Soc. B Biol. Sci..

[27] Torri L., Tuccillo F., Bonelli S., Piraino S., Leone A. (2020). The Attitudes of Italian Consumers towards Jellyfish as Novel Food. Food Qual. Prefer..

[28] De Zoysa M. (2012). Medicinal Benefits of Marine Invertebrates: Sources for Discovering Natural Drug Candidates. Adv. Food Nutr. Res..

[29] Youssef J., Keller S., Spence C. (2019). Making Sustainable Foods (Such as Jellyfish) Delicious. Int. J. Gastron. Food Sci..

[30] Kalina R.S., Peigneur S., Zelepuga E.A., Dmitrenok P.S., Kvetkina A.N., Kim N.Y., Leychenko E.V., Tytgat J., Kozlovskaya E.P., Monastyrnaya M.M. (2020). New Insights into the Type II Toxins from the Sea Anemone *Heteractis crispa*. Toxins.

[31] Liao Q., Feng Y., Yang B., Lee S.M.-Y. (2019). Cnidarian Peptide Neurotoxins: A New Source of Various Ion Channel Modulators or Blockers against Central Nervous Systems Disease. Drug Discov. Today.

[32] Augustin R., Anton-Erxleben F., Jungnickel S., Hemmrich G., Spudy B., Podschun R., Bosch T.C.G. (2009). Activity of the Novel Peptide Arminin against Multiresistant Human Pathogens Shows the Considerable Potential of Phylogenetically Ancient Organisms as Drug Sources. Antimicrob. Agents Chemother..

[33] Liang X., Wang R., Dou W., Zhao L., Zhou L., Zhu J., Wang K., Yan J. (2018). Arminin 1a-C, a Novel Antimicrobial Peptide from Ancient Metazoan Hydra, Shows Potent Antileukemia Activity against Drug-Sensitive and Drug-Resistant Leukemia Cells. Drug Des. Devel. Ther..

[34] Kelman D., Kashman Y., Rosenberg E., Kushmaro A., Loya Y. (2006). Antimicrobial Activity of Red Sea Corals. Mar. Biol..

[35] Lei J., Sun L.C., Huang S., Zhu C., Li P., He J., Mackey V., Coy D.H., He Q.Y. (2019). The Antimicrobial Peptides and Their Potential Clinical Applications. Am. J. Transl. Res..

[36] Palla F., Barresi G., Giordano A., Schiavone S., Trapani M.R., Rotolo V., Parisi M.G. (2016). Cold-Active Molecules for a Sustainable Preservation and Restoration of Historic-Artistic Manufacts. Int. J. Conserv. Sci..

[37] Barresi G., Di Carlo E., Trapani M.R., Parisi M.G., Chille C., Mule M.F., Cammarata M., Palla F. (2015). Marine Organisms as Source of Bioactive Molecules Applied in Restoration Projects. Herit. Sci..

[38] Stabili L., Schirosi R., Parisi M.G., Piraino S., Cammarata M. (2015). The Mucus of *Actinia equina* (Anthozoa, Cnidaria): An Unexplored Resource for Potential Applicative Purposes. Mar. Drugs.

[39] Bradford M.M. (1976). A Rapid and Sensitive Method for the Quantitation of Microgram Quantities of Protein Utilizing the Principle of Protein-Dye Binding. Anal. Biochem..

[40] Stabili L., Licciano M., Giangrande A., Gerardi C., De Pascali S.A., Fanizzi F.P. (2019). First Insight on the Mucus of the Annelid *Myxicola infundibulum* (Polychaeta, Sabellidae) as a Potential Prospect for Drug Discovery. Mar. Drugs.

[41] Balouiri M., Sadiki M., Ibnsouda S.K. (2016). Methods for in Vitro Evaluating Antimicrobial Activity: A Review. J. Pharm. Anal..

[42] Ballarin L., Cammarata M., Cima F., Grimaldi A., Lorenzon S., Malagoli D., Ottaviani E. (2008). Immune-Neuroendocrine Biology of Invertebrates: A Collection of Methods. Invertebr. Surviv. J..

[43] Wang Z., Wang G. (2004). APD: The Antimicrobial Peptide Database. Nucleic Acids Res..

[44] Thomas S., Karnik S., Barai R.S., Jayaraman V.K., Idicula-Thomas S. (2010). CAMP: A Useful Resource for Research on Antimicrobial Peptides. Nucleic Acids Res..

[45] Torrent M., Nogués V.M., Boix E. (2009). A Theoretical Approach to Spot Active Regions in Antimicrobial Proteins. BMC Bioinform..

[46] Torrent M., Di Tommaso P., Pulido D., Nogués M.V., Notredame C., Boix E., Andreu D. (2012). AMPA: An Automated Web Server for Prediction of Protein Antimicrobial Regions. Bioinformatics.

[47] White S.H., Wimley W.C. (1999). Membrane Protein Folding and Stability: Physical Principles. Annu. Rev. Biophys. Biomol. Struct..

[48] Waterhouse A., Bertoni M., Bienert S., Studer G., Tauriello G., Gumienny R., Heer F.T., de Beer T.A.P., Rempfer C., Bordoli L. (2018). SWISS-MODEL: Homology Modelling of Protein Structures and Complexes. Nucleic Acids Res..

[49] Studer G., Rempfer C., Waterhouse A.M., Gumienny R., Haas J., Schwede T. (2020). QMEANDisCo-Distance Constraints Applied on Model Quality Estimation. Bioinformatics.

